# Butyrate Improves the Metabolic Disorder and Gut Microbiome Dysbiosis in Mice Induced by a High-Fat Diet

**DOI:** 10.3389/fphar.2019.01040

**Published:** 2019-09-13

**Authors:** Feng Gao, Yi-Wei Lv, Jie Long, Jie-Mei Chen, Jiu-ming He, Xiong-Zhong Ruan, Hai-bo Zhu

**Affiliations:** ^1^State Key Laboratory for Bioactive Substances and Functions of Natural Medicines, Beijing Key Laboratory of New Drug Mechanisms and Pharmacological Evaluation Study, Institute of MateriaMedica, Chinese Academy of Medical Sciences & Peking Union Medical College, Beijing, China; ^2^John Moorhead Research Laboratory, Department of Renal Medicine, University College London Medical School, University College London, London, United Kingdom

**Keywords:** AMPK, GLUT4, metabolomics, sodium butyrate, 16s rRNA

## Abstract

**Background:** Metabolic syndrome (MS) is one of the major causes of coronary artery diseases (CAD). Gut microbiome diversity and its natural fermentation products are not only correlated with MS and CAD, but their correlations also appear to be stronger than the associations with traditional risk factors. Therefore, the aim of this study was to provide a new potential pathway for the natural fermentation product butyrate to improve MS and to examine whether it is associated with serum metabolic profiles and gut flora composition.

**Methods:** C57BL/6J mice fed a high-fat diet (HFD) were treated with 400 mg/kg of sodium butyrate for 16 weeks. Blood and fecal samples were collected, and the metabolite concentrations and 16s rRNA were measured with liquid chromatography–MS and Illumina platform, respectively. The plasma differential metabolites and gut microbiome composition were analyzed with XCMS online and QIIME 2, respectively.

**Results:** Gut microbiome-derived butyrate reduced glucose intolerance and insulin resistance, resisting HFD-induced increase in the relative abundance of f_*Lachnospiraceae*, f_*Rikenellaceae*, and f_*Paraprevotellaceae*. Meanwhile, sodium butyrate increased the levels of α-linolenate, all-trans-retinal, resolvin E1, and leukotriene in the plasma, and the differential pathways showed enrichment in mainly resolvin E biosynthesis, histidine degradation, lipoxin biosynthesis, and leukotriene biosynthesis. Moreover, sodium butyrate increased the levels of phosphorylated-adenosine 5′-monophosphate-activated protein kinase (p-AMPK) and facilitated glucose transporter member 4 (GLUT4) in the adipose tissue.

**Conclusion:** Butyrate can induce AMPK activation and GLUT4 expression in the adipose tissue, improving cardiovascular disease (CVD)-related metabolic disorder, resisting HFD-induced gut microbiome dysbiosis, and promoting resolvin E1 and lipoxin biosynthesis. Oral supplement of the natural fermentation product butyrate can be a potential strategy for preventing CVD.

## Introduction

Previous studies have indicated that the risk of developing cardiovascular disease (CVD) in patients with metabolic syndrome (MS) is approximately 2-fold higher than that in healthy people. Gut microbiome diversity is not only correlated with MS and CVD, but the correlations also appear to be stronger than the associations with traditional risk factors (Flego et al., 2016; Bogiatzi et al., 2018; Menni et al., 2018). Diet is an important factor affecting the diversity and composition of the gut microbiome, especially high-fat diet (HFD), which is not only associated with gut microbiome composition but is also a risk factor for MS and CVD (Daliri et al., 2018). However, how HFD affects MS and CVD via the gut microbiome still remains largely unknown.

Butyrate is a natural fermentation product of the gut, and it plays a crucial role in maintaining the homeostasis of host metabolism and gut microbiome diversity (Kasubuchi et al., 2015). A reduction in butyrate concentration in the gut is related to the development of the MS and CVD (Murugesan et al., 2018). Although oral butyrate supplement can improve HFD-induced MS and coronary artery disease in mice *via* histone deacetylases (Gao et al., 2009), a recent research indicated that oral butyrate treatment exerts a beneficial effect on glucose metabolism in healthy males instead of MS patients (Bouter et al., 2018), thus suggesting that the effects and molecular mechanism of butyrate in glucose metabolism and insulin resistance need to be confirmed further.

Butyrate is mostly produced in the intestinal epithelium, and its concentration is very low in the blood (Leonel and Alvarez-Leite, 2012). Therefore, butyrate-derived blood metabolites could be a potential pathway through which butyrate can regulate the physiological processes in the host. However, it has been rarely reported how butyrate impacts the metabolites in the blood. Comparative metabolomics based on the pathological process and circumstantial stimuli is an effective approach to discover the relationship between metabolites and pathways (Shan et al., 2018). 16s RNA sequencing is extensively applied to describe the gut microbiome profile.

Here, we used metabolomics and 16s RNA sequencing to identify the differential metabolites and gut microbiome related to MS, thus evaluating the effects of butyrate on glucose homeostasis and metabolic profiles. Discovery of a relationship between the natural fermentation product butyrate and MS provides opportunities to identify new strategies and targets for CVD.

## Materials and Methods

### Treatment of Animals

Male C57BL/6J mice (4 weeks old) were purchased from Beijing Vital River Laboratory Animal Technology Co., Ltd. All animals were housed in a temperature-controlled environment with a 12 h light/12 h dark cycle and allowed free access to food and water. The animal study was reviewed and approved by the Ethics Committee of Institute of Materia Medica, Chinese Academy of Medical Sciences & Peking Union Medical College. After 1 week of adaptation, 36 mice were randomly assigned to control, HFD, and HFD plus sodium butyrate group. Animals in the control group were fed with a normal diet and given daily gavage with water. Animals in the HFD group were fed with an HFD and given daily gavage with water (10% lard, 1.00% cholesterol, 0.4% sodium cholate, and 10% custard powder) (Wang et al., 2017).

Sodium butyrate (molecular formula: C_4_H_7_NaO_2_, molecular weight: 110.09, purity: 99%) was purchased from Shanghai Aladdin Biochemical Technology Co., Ltd. Animals in the sodium butyrate group were fed with HFD and given daily gavage with sodium butyrate (400 mg/kg, dissolved in water) for 16 weeks.

### Glucose Tolerance Tests and Glucose-Induced Insulin

Blood samples of C57BL/6J mice were collected from the caudal vein after fasting for 16 h. The concentration of blood glucose was measured with a blood glucose meter (Accu-ChekActive [Model GB], Roche Diabetes Care Gmbh, Mannheim, Germany). Glucose-induced insulin secretion was measured with an Insulin Kit [Insulin (Mouse) Ultrasensitive EIA, 96w, “RUO” Alpco].

### Measurement of Total Cholesterol, Triglyceride, and Glycosylated Protein

Blood samples of C57BL/6J mice were collected from the caudal vein after fasting for 12 h. Analyses of cholesterol (CHOD-PAP kit, BioSino Bio-Technology & Science Inc, Beijing, China), triglyceride (GPO-PAP kit, BioSino Bio-Technology & Science Inc, Beijing, China), and glycosylated protein (GSP-NBT, BioSino Bio-Technology & Science Inc, Beijing, China) were performed by using the standardized kits.

### Serum Metabolomics

The metabolites in serum were determined by a Dionex UHPLC Ultimate 3000 system (Thermo Scientific, Dionex, Sunnyvale, CA, USA) coupled to a Q-Exactive mass spectrometer. Blood samples were collected at week 4. A quantity of 50 μL serum was precipitated with 150 μL acetonitrile. The mixture was vortexed for 300 s at 2500 rpm and then centrifuged at 10,000 rpm under 4°C for 5 min. The supernatant was collected and dried under nitrogen gas flow. The residues were dissolved in 100 μL acetonitrile:H_2_O (2:98, V/V) for liquid chromatography–MS analysis. Chromatographic separation was performed on a Waters HSS T3 (C18) column (2.1 × 100 mm, 1.8 mm), and the column temperature was maintained around 35°C. The mobile phase consisted of 0.1% formic acid (A) and acetonitrile (B), and the flow rate was 250 μL/min. The injection volume was 10 μL. The gradient conditions were as follows: 0 min, 2% B; 9 min, 60% B; 18 min, 60% B; 20 min, 100% B; and 30 min, 100% B. The mass spectrometric settings for positive/negative ion modes were as follows: scan mode, full MS; scan range, m/z 100–1000; resolution, 70,000; automatic gain control target, 3 × 106; maximum IT, 100 ms; spray voltage (+/−), 3.5/3.2 kV; capillary temperature, 320°C; sheath gas flow rate (+/−), 40/7; and aux gas flow rate (+/−), 11/0. The non-linear alignment of data in the time domain and the spontaneous integration and isolation of peak intensities were performed by using XCMS online. Partial least squares discriminant analysis (PLS-DA), principal components analysis (PCA), principal coordinates analysis (PCoA), and volcano plot were performed using the Omicshare platform.

### 16s RNA Sequencing of Fecal Samples

Fecal samples from C57BL/6J mice were collected and stored at −80°C at week 4. The V3–V4 hypervariable domain of the 16S rRNA gene was ampliﬁed with the specific primers 341F: (5′-CCTAYGGGRBGCASCAG-3′ and 806R (5′-GGACTACNNGGGTATCTAAT-3′) (**Supplementary Material**). Sequencing was performed using a single-end conﬁguration by an Illumina sequencing platform. Phenotypic analysis was carried out by QIIME2 (version: 2018.11). The sequence quality control and feature table construction were performed with the DADA2 plugin. Phylogenetic diversity analyses were performed with q2-phylogeny and q2-diversity. The feature classifiers were trained by q2-feature-classifier within QIIME2. The box plot was generated using the Omicshare platform.

### Western Blot Assay

Proteins were prepared from tissues and cells in radio immunoprecipitation assay (RIPA) lysis buffer with protease inhibitors and phosphatase inhibitor cocktail (Roche, Switzerland). Then, SDS-polyacrylamide gel electrophoresis (SDS-PAGE) was conducted, and bands were transferred to polyvinylidene fluoride (PVDF) membrane, followed by incubation with primary antibodies against phospho-AMPK (phosphorylated-adenosine 5′-monophosphate-activated protein kinase), phospho-ACC, phospho-LKB1 (Ser428), Glut4, GAPDH, and di-methyl-histone H3(Lys27) (diluted at 1:1000, catalog number: 3033, 3661, 3482, 2213, 5174, and 9728, respectively, Cell Signaling Technology, Boston, MA, USA). Corresponding secondary antibodies (diluted at 1:5000) were added, and bands were visualized with enhanced chemiluminescence reagents (Thermo Fisher Scientific, Waltham, MA, USA). Signals were normalized to those of β-actin (diluted at 1:5000, catalog number: 4967, Cell Signaling Technology, Boston, MA, USA).

### Hematoxylin–Eosin Staining

Sections (3 μm) were obtained from each paraffin block using a microtome and stained with hematoxylin–eosin. Samples were immersed in xylene and alcohol, stained with hematoxylin for 5 min, stained with eosin for 3 min, and re-immersed in alcohol and xylene.

## Results

### HFD Induces MS in Mice

**Figure 1:**
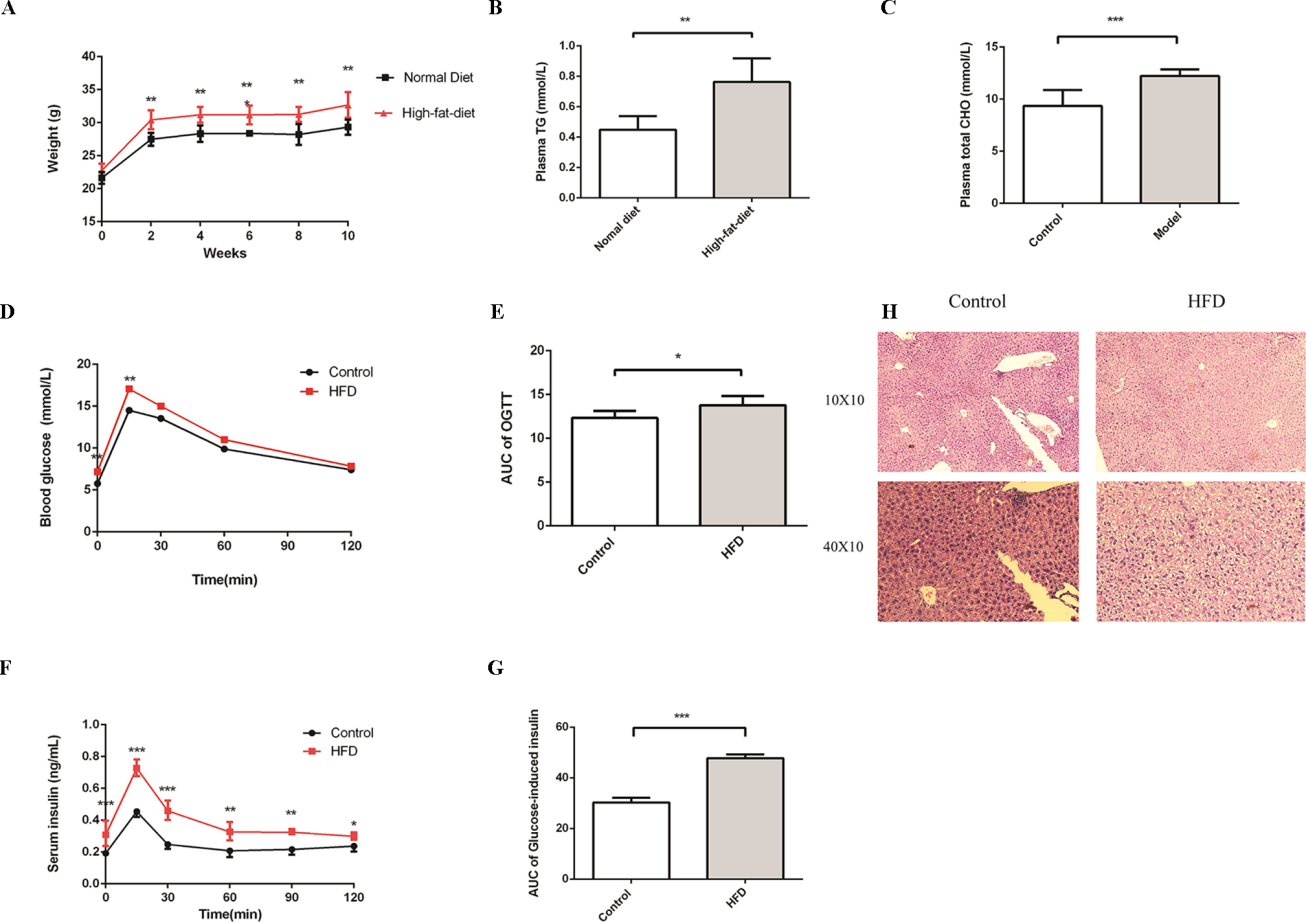
HFD induces metabolic syndrome in C57BL/6J mice. Male C57BL/6J mice were allowed free access to the high-fat and normal diet. **(A)** The body weight of the HFD group was significantly higher compared to the control group. **(B** and **C)** Compared to the control group, the levels of plasma TG and Total CHO were significantly increased in the HFD group at week4 (n = 6, *p < 0.05). **(D** and **E)** After fasting for16 hours, mice received i.p. injections of 1 g/kg glucose. Blood glucose level was measured at 15, 30, 60, and 120 min. Compared to the control group, the level of blood glucose was significantly increased in the HFD group at 0 and 15 min (n = 5, *p < 0.05). Data are representative of five mice in each case. Data represent mean ± sem. *p < 0.05; **p < 0.01; ***p < 0.001 via unpaired t-test at each time point. **(F** and **G)** After fasting for16 hours, mice received i.p. injections of 1 g/kg glucose. Serum insulin levels were measured at 15, 30, 60, 90, and 120 min. Compared to the control group, the level of serum insulin was significantly increased in the HFD group (n = 5, *p < 0.05). Data are representative of five mice in each case. Data represent mean ± sem. *p < 0.05; **p < 0.01; ***p < 0.001 via unpaired t-test at each time point. **(H)** Compared to the control group, the number of cavities was increased in the HFD group.

To evaluate the impact of HFD on the homeostasis of glucose and lipids, we treated C57BL/6J male mice with HFD for 16 weeks. At the beginning of the experiment, mice were randomly divided into control and HFD groups. The levels of plasma glucose, total cholesterol, and triglycerides, which can characterize MS, were detected at week 4. As expected, compared to the control group, the weight was much higher in the HFD group (**Figure 1A**). In addition, we observed an apparent increase of triglyceride and total cholesterol in the HFD group at week 4 (**Figures 1B, C**), glucose intolerance and insulin resistance appeared at week 16 (**Figures 1D–G**). In the control group, hepatocytes were arranged radially around a vein in the center and hepatic structure was clearly visible. Few small-sized cavities were found, whereas in the HFD group many small-sized cavities were found (**Figure 1H**). These results indicated that HFD impaired the homeostasis of glucose and lipids, which is in agreement with previous studies (Araujo et al., 2011).

### HFD Changes the Metabolic Profiles in Mice

**Figure 2:**
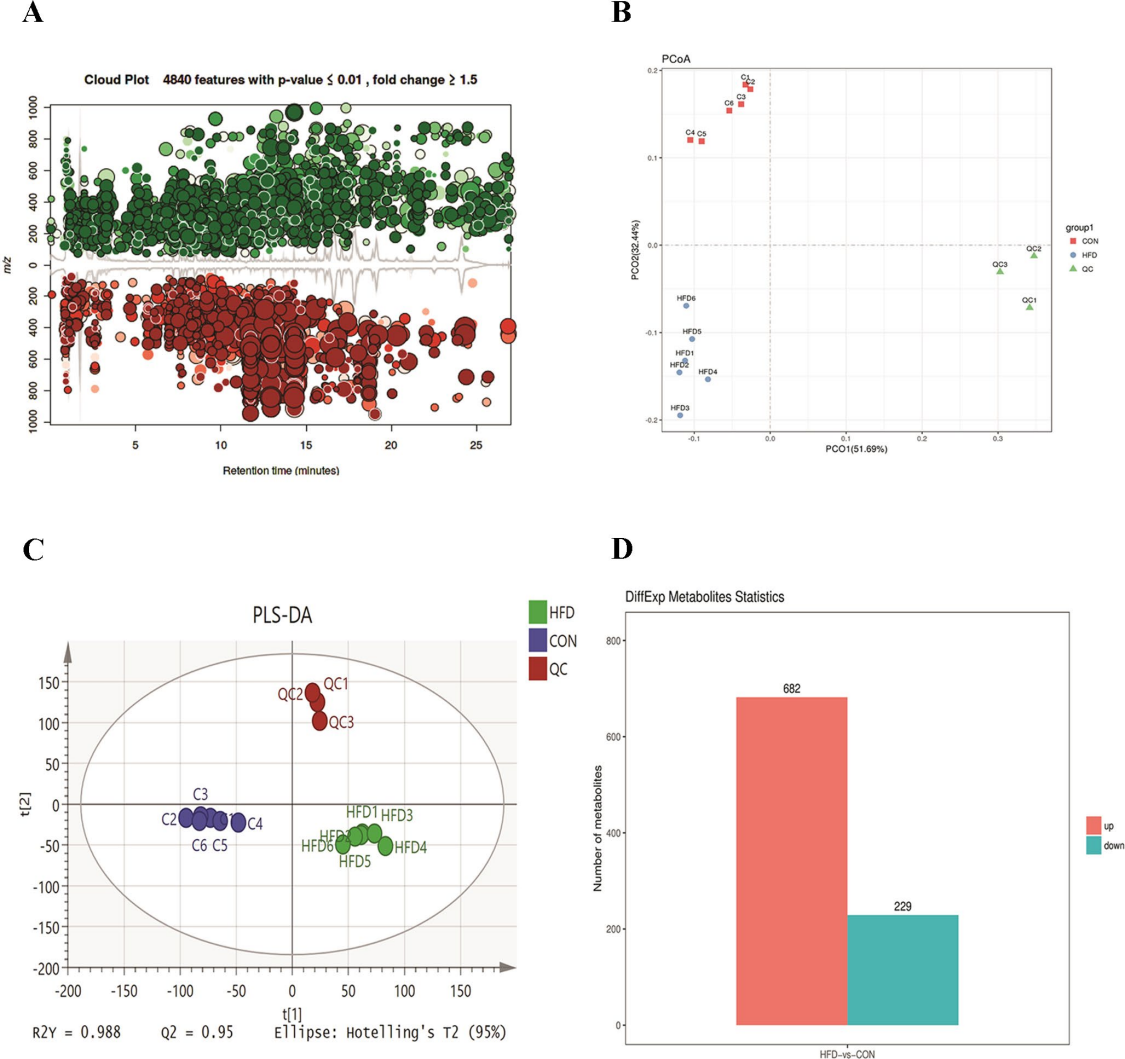
HFD changes metabolic profiles in C57BL/6J mice. Blood samples were collected at week 4. Serum metabolomics analysis was performed with the pairwise method on the XCMS online platform. **(A)** Differential featured plots were shown. Only dysregulated features (p-value ≤ 0.05 and fold change ≥ 1.5) are displayed. Up-regulated features are shown in green, and down-regulated features are shown in red. **(B)** PCoA of the features intensities from all simples. The control group is shown in red; the HFD group is shown in blue; the quality control is shown in green **(C)** PLS-DA scores plot of the control and HFD group. Blue spots represent the control group, and the red spots represent the HFD group. **(D)** The red column represents up-regulated metabolites, and green column represents down-regulated metabolites in the HFD group.

**Figure 3:**
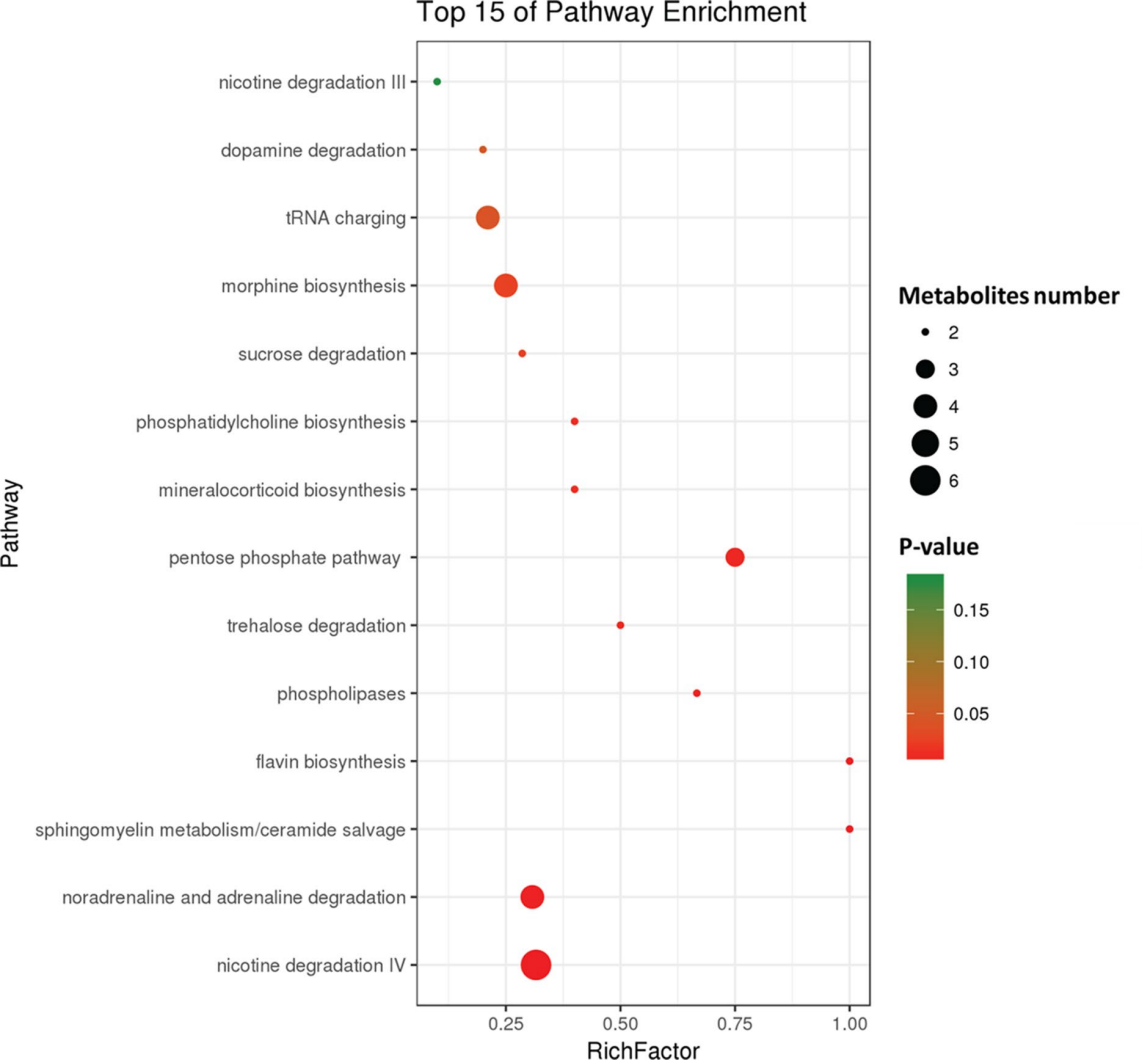
Differential pathway analysis of the HFD and control group. The radius of each circle represents the number of metabolites relative to the number of metabolites represented by other circles. The colors from green to red indicate the elevated p-value.

To further investigate the change in metabolic profiles induced by HFD, we performed serum untargeted metabolomics using a liquid chromatograph–mass spectrometer. As shown by the metabolomics cloud plot (**Figure 2A**), a total of 4840 features were observed (p-value ≤ 0.05 and fold ≥ 1.5). The PCoA analysis displayed that there was a separate tendency of serum metabolites between the control and HFD groups (**Figure 2B**). The parameter values of R2 and Q2 from PLS-DA analysis were 0.988 and 0.95, respectively, indicating that this model showed excellent performance on predictive ability (**Figure 2C**). The values of variable importance in projection (VIP) above 1.0 and p-values below 0.05 were employed to identify significant metabolites related to group separation. Compared to the control group, a total of 682 metabolites were increased and 229 metabolites were decreased in the HFD group (**Figure 2D**). Differential pathways showed enrichment in nicotine degradation IV, noradrenaline and adrenaline degradation, sphingomyelin metabolism or ceramide salvage, phospholipases, and pentose phosphate pathway (**Figure 3**).

### Gut Flora Composition Is Associated With Plasma Biomarker and Metabolic Profiles

**Figure 4:**
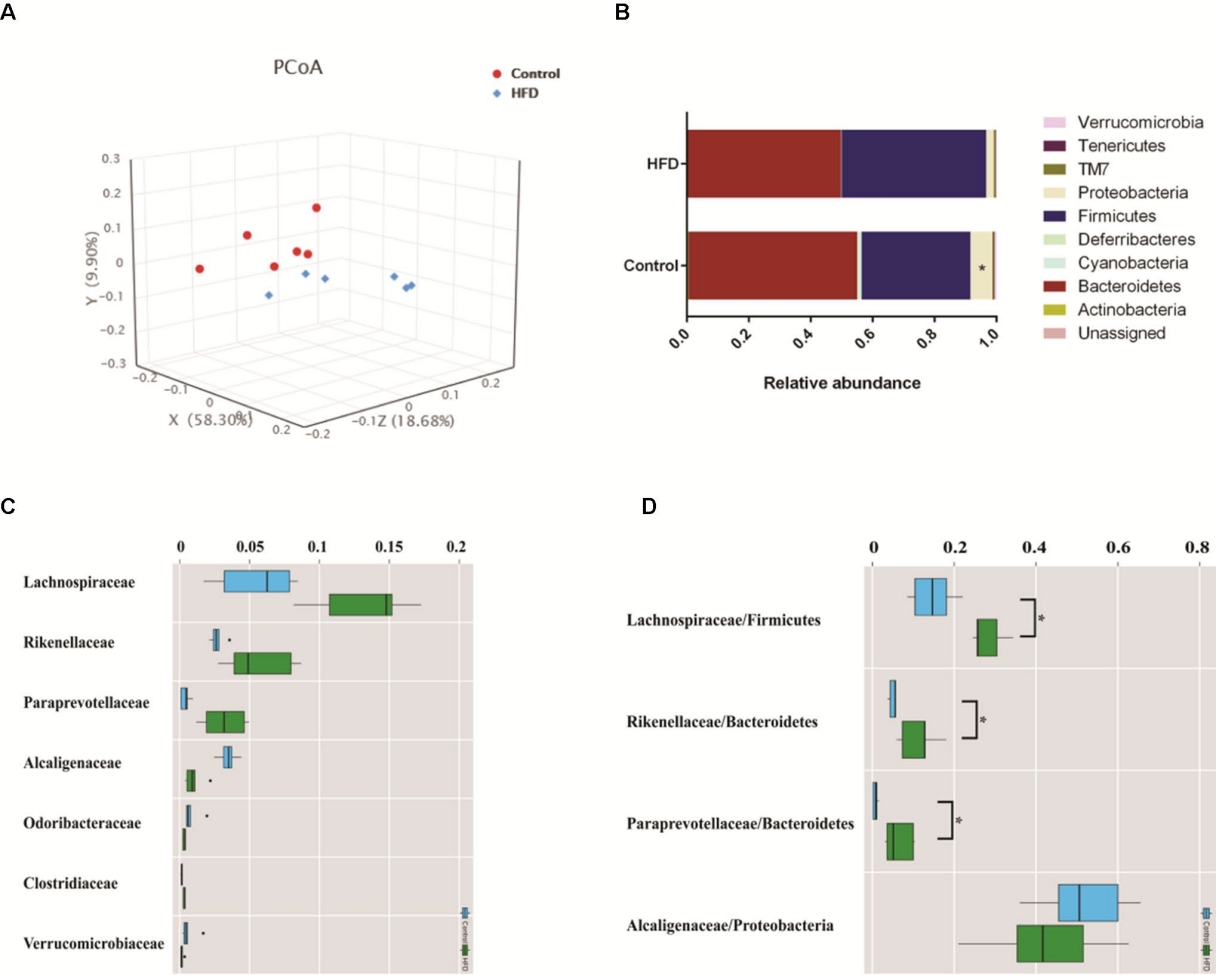
HFD alters the composition of gut microbiome in C57BL/6J mice. Fecal samples were collected at week 4. Fecal DNA was subjected to 16S rRNA sequencing and analyzed with QIIME2. (version 2018.11). **(A)** Principal coordinates analysis (PCoA) plot of Bray–Curtis distance. Red spots represent samples from the control group, and blue spots represents samples from the HFD group. **(B)** The relative abundance of a major microbial phylum in the control and HFD group. Compared to the HFD group, proteobacteria was a higher abundance in the control group. Data are mean ± SD (*p < 0.05; Wilcoxon matched-pairs signed rank test and Mann–Whitney U test). **(C)** Relative abundance of a major microbial family in the control and HFD group. The seven abundant families are shown. Data are mean ± SD (*p < 0.05; Wilcoxon matched-pairs signed rank test and Mann–Whitney U test). **(D)** The ratio of the major microbial family/phylum in the control and HFD group. Data are mean ± SD (*p < 0.05; Wilcoxon matched-pairs signed rank test and Mann–Whitney U test).

To investigate how HFD changes the gut microbiome composition, we analyzed fecal samples of mice in the HFD and control groups using 16S rRNA gene sequencing (Kovatcheva-Datchary et al., 2015). PCoA based on the individual Bray–Curtis distance indicated that there was a significant separation tendency between these two groups (**Figure 4A**). Although p_Bacteroidetes and p_Firmicutes were the dominant bacteria at the phylum level, our results indicated that there was no significant difference between these two groups (**Figure 4B**). At the family level, we observed an increased relative abundance of f_Lachnospiraceae, f_Rikenellaceae, and f_*Paraprevotellaceae* and a decreased relative abundance of f_*Alcaligenaceae* in the HFD group (**Figure 4C**). Meanwhile, the ratios of f_*Lachnospiraceae*/p_*Firmicutes*, f_*Rikenellaceae*/p_*Bacteroides*, and f_*Paraprevotellaceae*/p_*Bacteroides* were also increased, suggesting that p_*Lachnospiraceae*, p_*Rikenellaceae*, and p_*Paraprevotellaceae* were the dominant bacteria in the HFD group and were associated with HFD-induced MS (**Figure 4D**).

**Figure 5:**
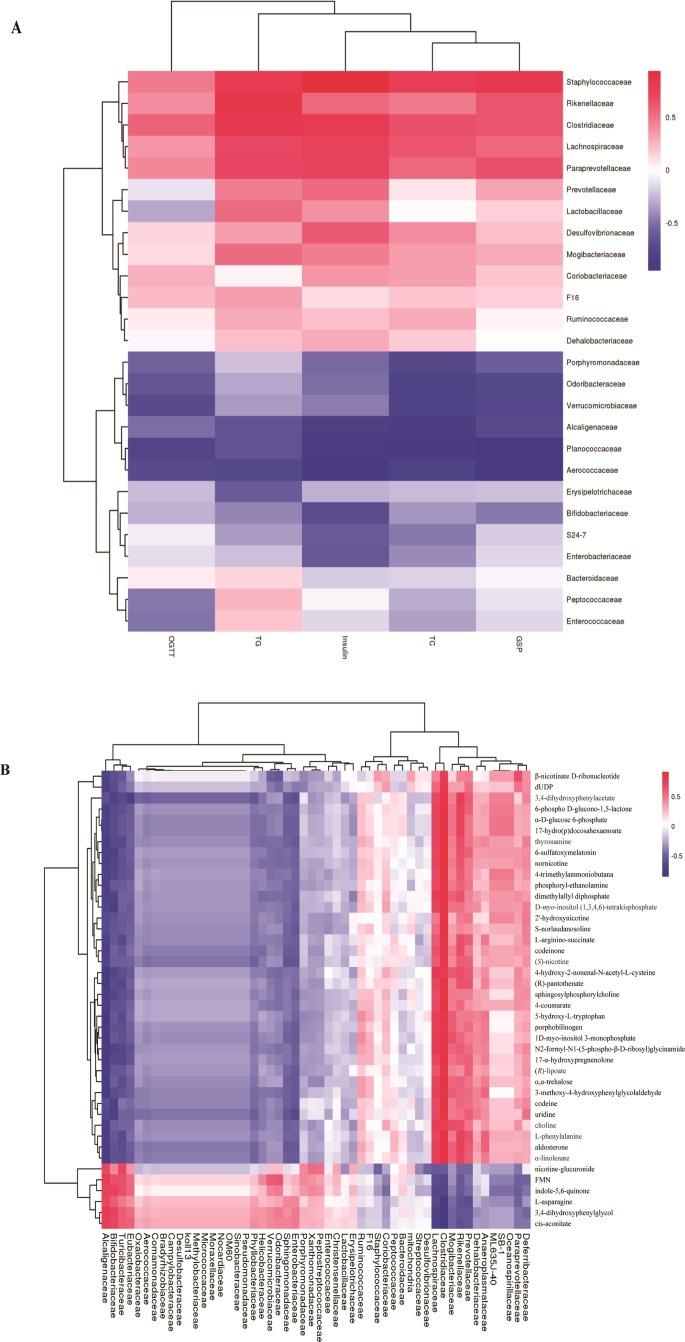
Heatmap of gut microbiome, plasma biomarker, and plasma metabolites. **(A)** Pearson correlation of plasma biomarker and gut microbiome.**(B)** Pearson correlation between metabolites and gut microbiome. The colors from blue to red indicate the elevated value of correlation coefficient.

To explore whether the alteration of gut composition is associated with plasma biomarkers and metabolic profiles, we performed a Pearson correlation analysis. The results are displayed in **Figure 5**, and they showed that relative abundance of f_*Lachnospiraceae*, f_*Rikenellaceae*, and f_*Paraprevotellaceae* had a positive correlation with the levels of total cholesterol, triglyceride, and glucose resistance, and a negative correlation with glucose tolerance. Moreover, f_*Lachnospiraceae*, f_*Rikenellaceae*, and f_*Paraprevotellaceae* relative abundances were associated with MS-related metabolites, including choline and L-argininosuccinate. Therefore, we deduced that gut composition is associated with metabolic profiles and plasma biomarkers, suggesting that gut composition could affect MS via metabolites.

### Butyrate Improves HFD-Induced MS in Mice

**Figure 6:**
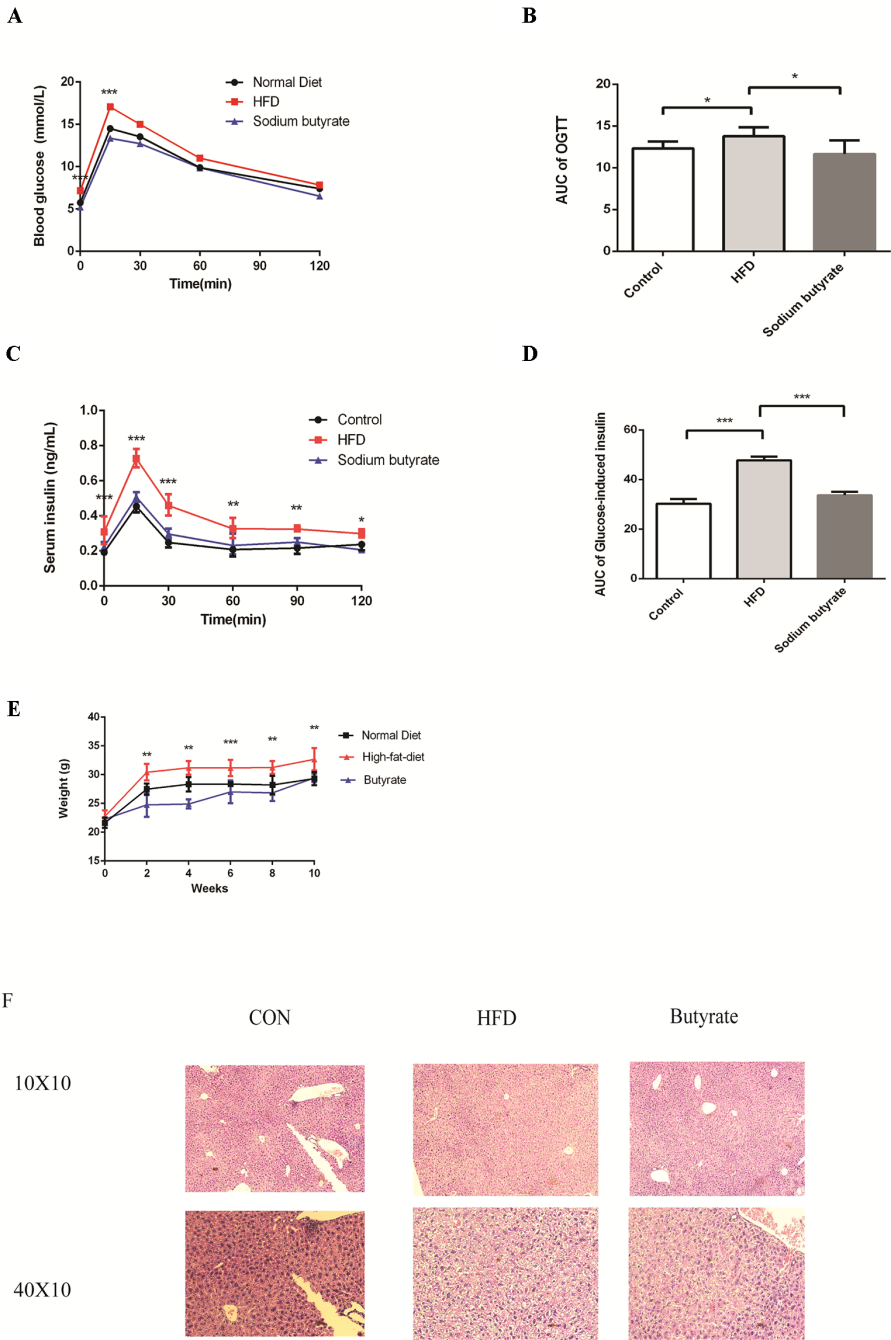
Butyrate improves HFD-induced metabolic syndrome in C57BL/6J mice. Male C57BL/6J mice were divided into the HFD group and Butyrate group. Butyrate group was oral gavaged sodium butyrate (400 mg/kg) for 16 weeks. **(A** and **B)** After 16 hours of fasting, mice received i.p. injections of 1 g/kg glucose. Blood glucose levels were measured at 15, 30, 60, and 120 min. Compared to the HFD group, blood glucose level was significantly reduced at 0 and 15 min in the butyrate group (n = 5, *p < 0.05). Data are representative of five mice in each case. Data represent mean ± sem. *p < 0.05; **p < 0.01; ***p < 0.001 *via* unpaired t-test at each time point. **(C** and **D)** After 16 hours of fasting, mice received i.p. injections of 1 g/kg glucose. Serum insulin level was measured at 15, 30, 60, 90, and 120 min. Compared to the HFD group, serum insulin level was significantly reduced in the butyrate group (n = 5, *p < 0.05). Data are representative of five mice in each case. Data represent mean ± sem. *p < 0.05; **p < 0.01; ***p < 0.001 *via* unpaired t-test at each time point. **(E)** Body weight of mice on the high-fat diet was significantly higher than the butyrate group for 16 weeks. **(F)** Compared to the HFD group, few cavities were observed in the butyrate group.

To further determine the effects of butyrate on HFD-induced MS, we performed daily gavage of 6-week-old C57BL/6J mice fed an HFD with sodium butyrate (400 mg/kg) for 16 weeks. The levels of total cholesterol and triglyceride showed no significant difference between the sodium butyrate and HFD group, whereas HFD-induced glucose intolerance and insulin resistance were significantly reduced (**Figures 6A–D**). Compared to the HFD group, weight gain was alleviated by sodium butyrate (**Figure 6E**). In the control group, few small-sized cavities were found in hepatocytes, whereas in the HFD group large-sized cavities were observed. Compared to the HFD group, the number of cavities was reduced in the butyrate group (**Figure 6F**). These data demonstrated that butyrate could improve HFD-induced glucose metabolism disorder instead of lipid metabolism disorder.

**Figure 7:**
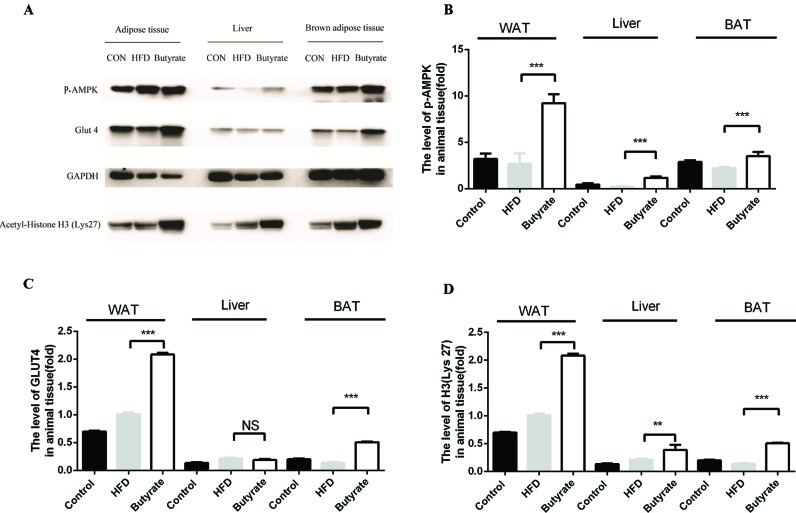
Sodium butyrate supplement increases the expression of p-AMPK, Glut4, and H3 (Lys 27) in adipose tissue from C57BL/6J mice. Murine tissues were collected after sodium butyrate treatment for 16 weeks **(A)** The western blotting of p-AMPK, Glut4, and H3(Lys 27). **(B, C,** and **D)** Compared to the HFD group, the levels of p-AMPK, Glut4, and H3 (Lys 27) were increased in the sodium butyrate group (p < 0.05). Data represent mean ± sem. *p < 0.05; **p < 0.01; ***p < 0.001 *via* unpaired t-test.

Given the effect of butyrate on glucose tolerance and insulin resistance, we examined the levels of p-AMPK and glucose transporter member 4 (GLUT4) in the liver and adipose tissue. As shown in **Figure 7**, sodium butyrate at 400 mg/kg increased the levels of p-AMPK, GLUT4, and histone acetylation in the white adipose tissue and brown adipose tissue from C57 BL/6J mice.

### Butyrate Alters the Metabolic Profiles in Mice

**Figure 8:**
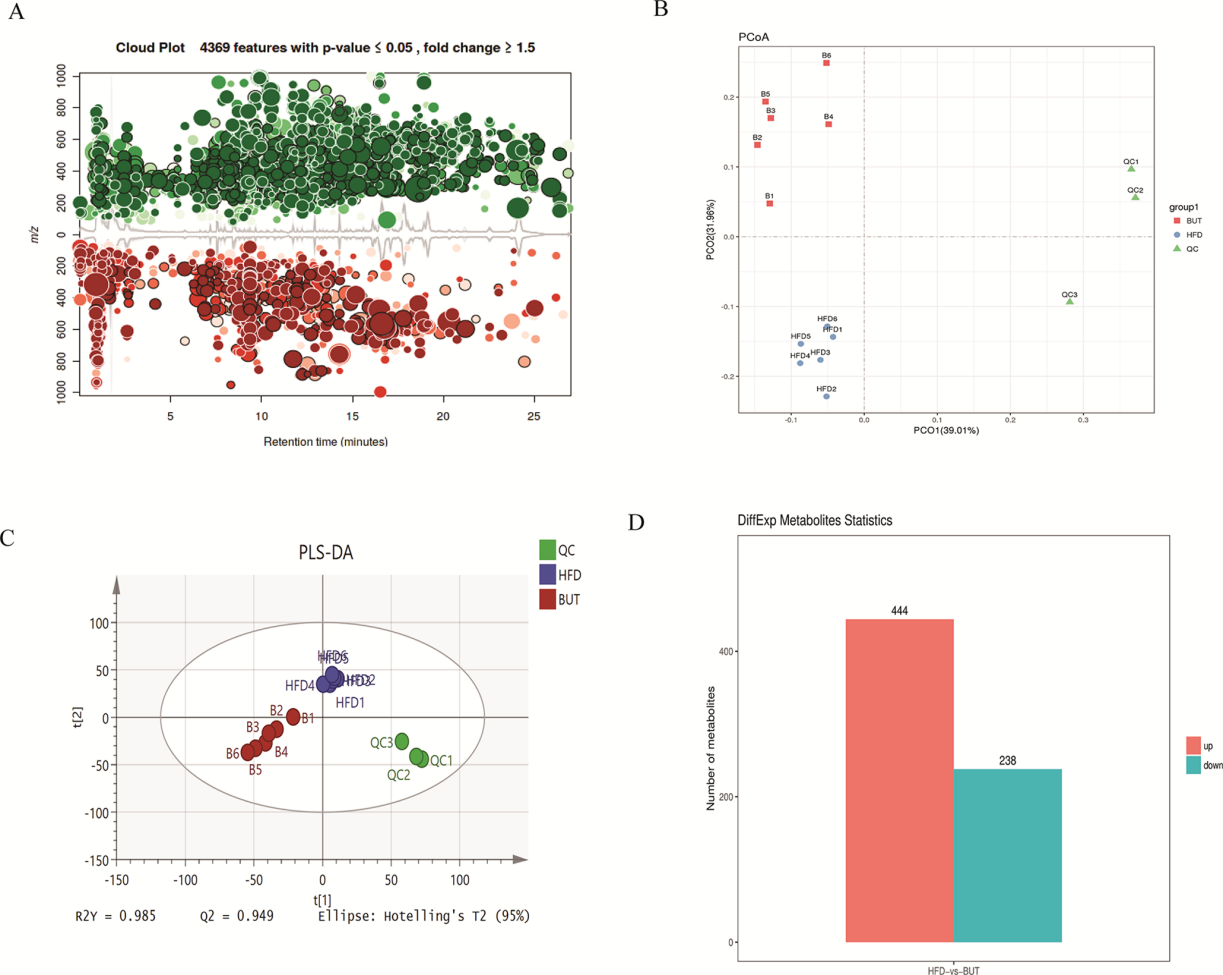
Sodium butyrate alters metabolic profiles in C57BL/6J mice. Blood samples were collected at week 4. Serum metabolomics analysis was performed with the pairwise method on the XCMS online platform. **(A)** Differential featured plots were shown. Only dysregulated features (p-value ≤ 0.05 and fold change ≥ 1.5) are displayed. Up-regulated features are shown in green, and down-regulated features are shown in red. **(B)** PCoA of the features intensities from all simples. HFD group is shown in blue; butyrate group is shown in red; quality control group is shown in green. **(C)** PLS-DA scores plot of the butyrate and HFD group. Red spots represent the butyrate group; blue spots represent the HFD group; green spots represent the quality control group. **(D)** The red column represents up-regulated metabolites, and the green column represents down-regulated metabolites in the HFD group.

**Figure 9:**
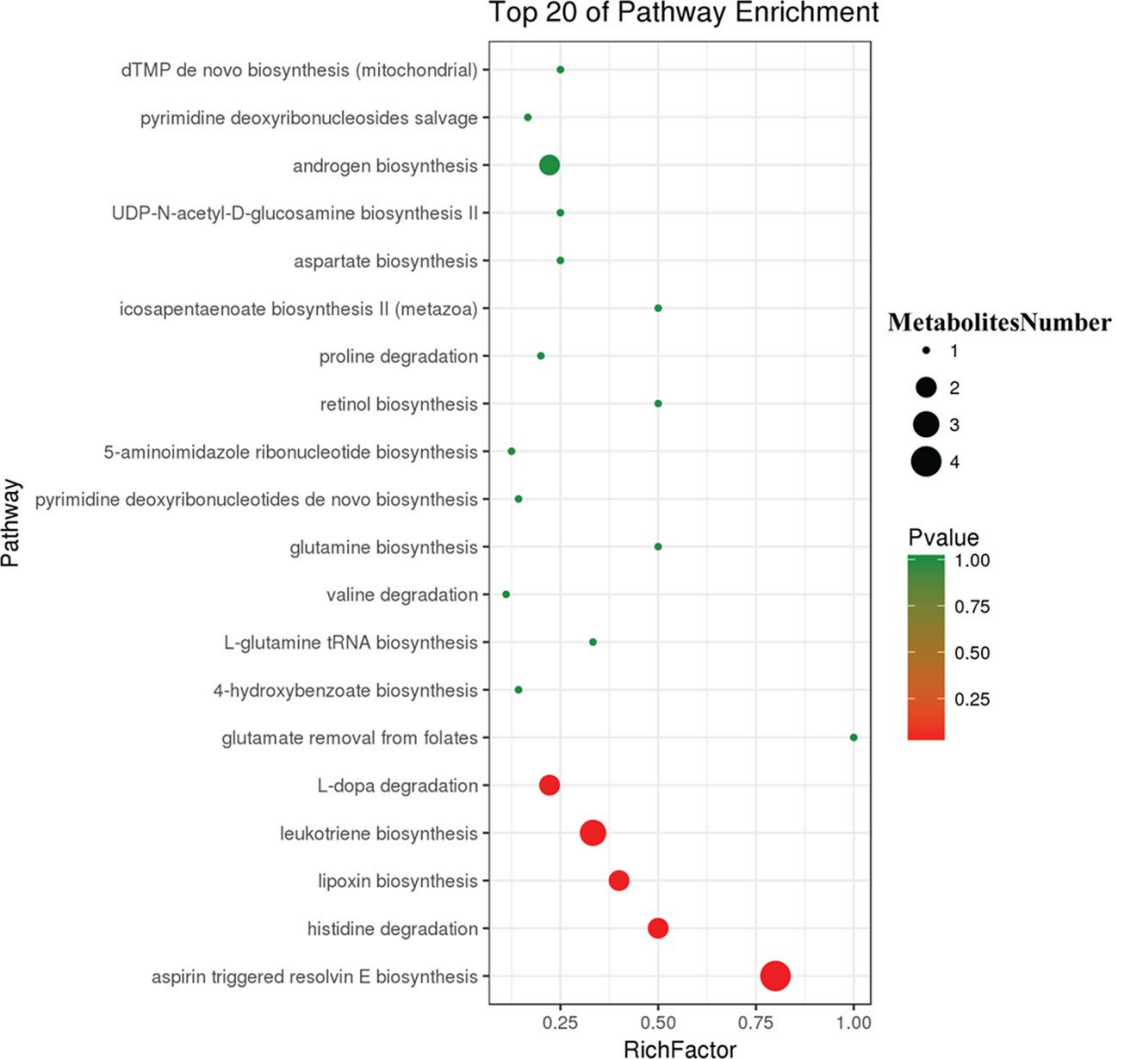
Differential pathway analysis of the HFD and butyrate group. The radius of each circle represents the number of metabolites relative to the number of metabolites represented by other circles. The colors from green to red indicate the elevated p-value.

To further explore the effect of butyrate on HFD-induced MS, we used serum untargeted metabolomics to monitor the change in the metabolic profile induced by sodium butyrate. As shown in the metabolomics cloud plot (**Figure 8A**), we observed a total of 4369 features (p-value ≤ 0.05 and fold ≥ 1.5). The PCoA analysis displayed that there was a separate tendency of serum metabolites between the sodium butyrate and HFD groups (**Figure 8B**). The parameter values of the R2 and Q2 from PLS-DA analysis were 0.985 and 0.949, respectively (**Figure 8C**). Compared to the control group, a total of 229 metabolites were increased and 444 metabolites were decreased in the sodium butyrate group (**Figure 8D**). Differential pathways showed enrichment in resolvin E biosynthesis, histidine degradation, lipoxin biosynthesis, and leukotriene biosynthesis (**Figure 9**).

### Butyrate-Mediated Metabolic Profile Alteration Is Associated With the Gut Microbiome

**Figure 10:**
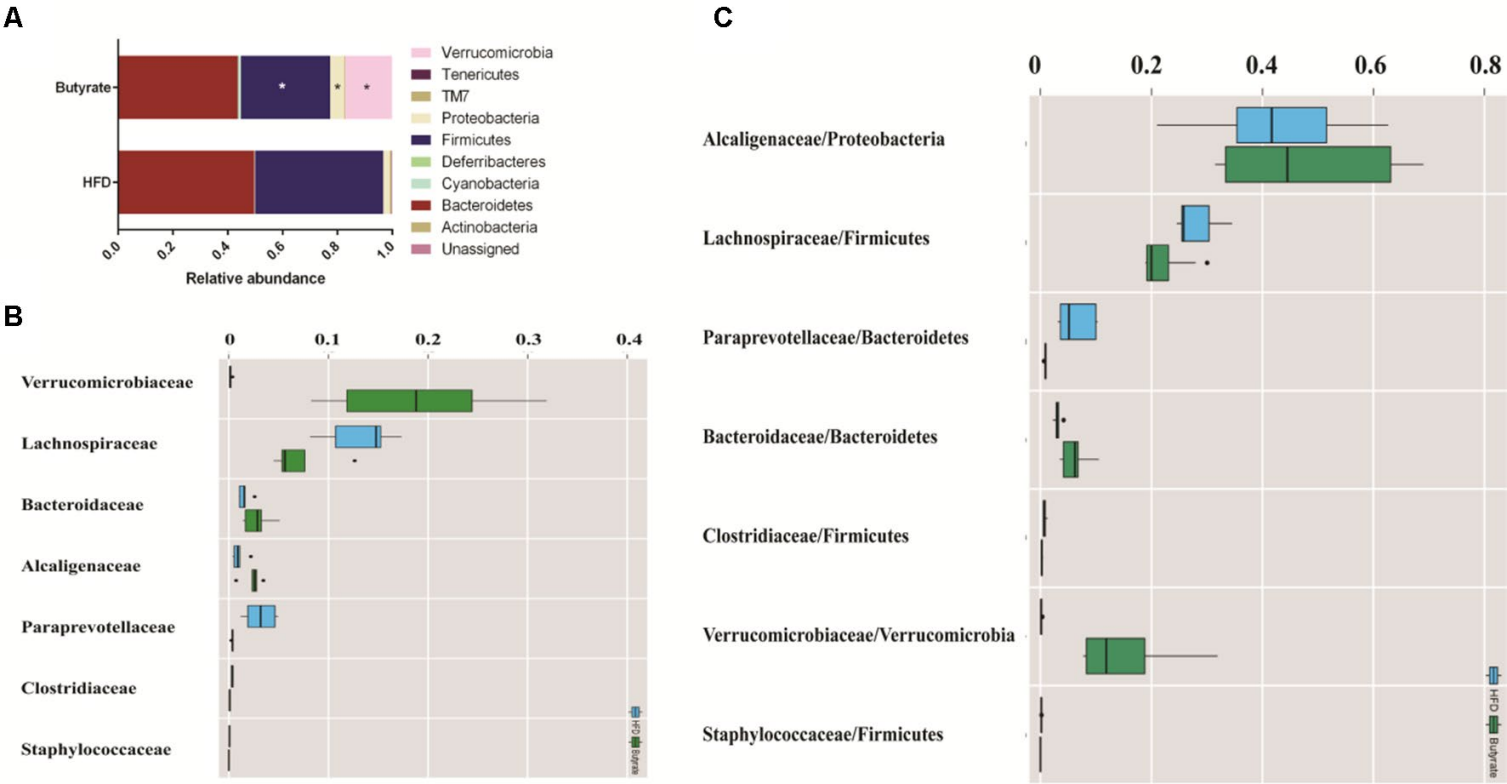
Butyrate resists the gut flora perturbation of C57BL/6J mice induced by HFD. Fecal samples from both HFD and butyrate group were collected at 4 weeks. Fecal DNA was subjected to 16S rRNA sequencing, and sequences were analyzed using QIIME2 (version 2018.11). **(A)** Relative abundance of a major microbial phylum in the butyrate and HFD group. Data are mean ± SD (*p < 0.05; Wilcoxon matched-pairs signed rank test and Mann–Whitney U test). **(B)** Relative abundance of a major microbial family in the butyrate and HFD group. The seven abundant families are shown. Data are mean ± SD (*p < 0.05; Wilcoxon matched-pairs signed rank test and Mann–Whitney U test). **(C)** The ratio of the major microbial family/phylum in the butyrate and HFD group. Data are mean ± SD (*p < 0.05; Wilcoxon matched-pairs signed rank test and Mann–Whitney U test).

**Figure 11:**
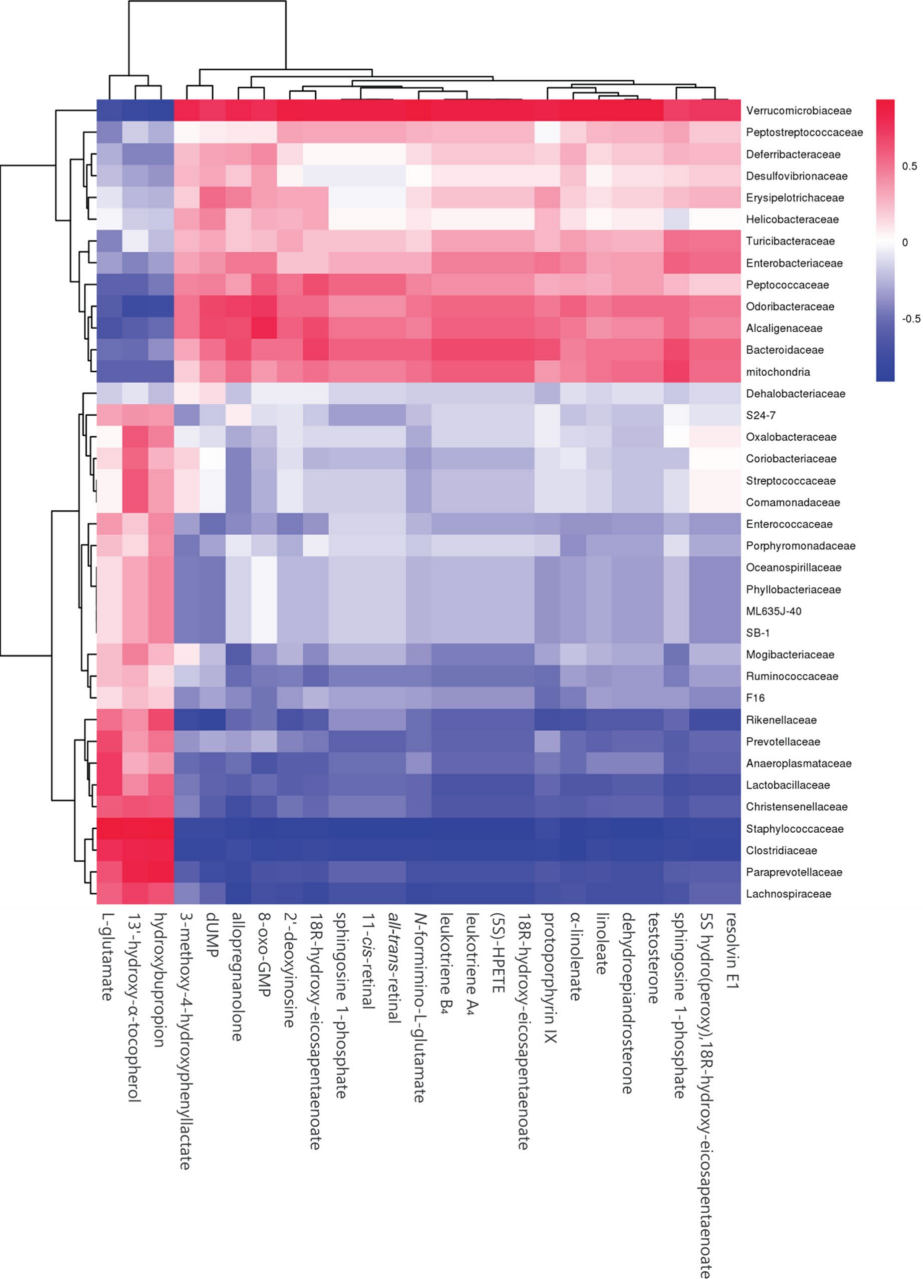
Heatmap of plasma metabolites and gut microbiome. The colors from blue to red indicate the elevated value of the correlation coefficient.

To further determine the effect of butyrate on gut microbiome composition, we performed daily gavage of 6-week-old C57BL/6J mice with sodium butyrate (400 mg/kg) and collected fecal samples at week 4. Results are displayed in **Figure 10**, and they showed that sodium butyrate increased the relative abundance of p_*Verrucomicrobia* and decreased the relative abundance of p_*Firmicutes*. At the family level, the relative abundances of f_*Verrucomicrobiaceae*, f_*Bacteroidaceae*, and f_*Alcaligenaceae* were increased and the relative abundances of f_*Lachnospiraceae* and f_*Paraprevotellaceae* were decreased in the butyrate group. The ratios of f_*Lachnospiraceae*/f_*Firmicutes* and f_*Paraprevotellaceae*/f_*Bacteroidetes* were decreased, suggesting that butyrate can resist HFD-induced increase in f_*Lachnospiraceae* and f_*Paraprevotellaceae*. Subsequently, we performed a Pearson correlation analysis to confirm whether there is a correlation between the gut microbiome and butyrate-mediated metabolic profile alteration. The results are shown in **Figure 11**, and they showed that f_*Verrucomicrobia* was negatively associated with L-glutamine, 13-hydroxy-α-tocopherol, and hydroxy-bupropion, and it was positively correlated with resolvin E 1, (5)-HPETE, and linoleate. The pattern in f_*Lachnospiraceae* and f_*Paraprevotellaceae* was opposite to that in f_*Verrucomicrobia*.

## Discussion

MS is a cluster of metabolic disorders, including central adiposity with visceral fat accumulation, dysglycemia, insulin resistance, and dyslipidemia (Sherling et al., 2017). The risk of developing CVD in patients with MS is approximately 2-fold higher than that in healthy people (Samson and Garber, 2014). Gut flora composition and microbiome-derived metabolites are correlated with CVD and appear to have a strong correlation than traditional risk factors (Mazidi et al., 2016). Accumulating studies have suggested that HFD-induced gut flora dysbiosis is related to MS and CVD (Murphy et al., 2015).

Here, we found that C57BL/6J mice fed an HFD not only showed altered gut flora diversity but also increased relative abundances of f_*Lachnospiraceae*, f_*Rikenellaceae*, and f_*Paraprevotellaceae* and decreased relative abundance of f_ *Alcaligenaceae*. The increase in the above-mentioned bacteria had a positive correlation with total cholesterol, triglyceride, and insulin resistance, and a negative correlation with glucose tolerance. Meanwhile, the increase in f_*Lachnospiraceae*, f_*Rikenellaceae*, and f_*Paraprevotellaceae* was associated with plasma metabolite change. These data demonstrated that HFD-induced gut flora dysbiosis was associated with plasma biomarker and plasma profile. Previous studies have also supported the claim that f_*Lachnospiraceae* and f_*Rikenellaceae* are related to glucose metabolism disorder and insulin resistance (Brown and Hazen, 2015; Brown and Hazen, 2018). Our results also showed that f_*Lachnospiraceae* and f_*Rikenellaceae* are associated with plasma metabolites including choline and branched chain amino acids (BCAAs). Previous studies have displayed that choline-derived trimethylamine oxide (TMAO) can stimulate the macrophages to engulf ox-LDL and enhance platelet hyperreactivity, ultimately promoting atherosclerosis progression. Elevated levels of BCAAs selectively disrupt mitochondrial pyruvate utilization and promote MS, whereas promoting BCAA catabolism or normalizing glucose utilization by overexpressing GLUT1 in the heart can rescue the metabolism disorder (Li et al., 2017).

HFD-induced microbiome dysbiosis is a major cause of butyrate reduction in the gut and is related to MS. Butyrate is a major metabolite from microbial fermentation in the gut (Duncan et al., 2002; Khan and Jena, 2015). As an important energy source for the intestinal epithelium and intestinal epithelial cells, butyrate not only can maintain physiological function but also can directly regulate gene expression and physiological process via inhibiting histone deacetylation (Perry et al., 2016; Menni et al., 2018). Although a probiotic supplement can increase the butyrate level, probiotics can occasionally cause detrimental metabolic activities or produce host deleterious metabolites and cause inappropriate immune responses and systemic infections (Daliri et al., 2018). Therefore, oral butyrate supplement is a better way to increase the butyrate level in the gut. In our study, we observed that oral sodium butyrate supplement could reduce HFD-induced glucose metabolites and insulin resistance, in agreement with previous studies (Si et al., 2018). However, we did not observe a significant effect of sodium butyrate on lipid metabolism in C57BL/6J mice.

AMPK is an important energy sensor in mammalian cells, and it can sense glucose via fructose-1,6-bisphosphate and aldolase. AMPK activation can increase glucose uptake, glycolysis, and mitochondrial biogenesis to improve glucose metabolism disorder and atherosclerosis (Kim et al., 2016). GLUT4 is a member of glucose transporter protein family and is expressed primarily in skeletal muscle and adipose tissue. The primary function of GLUT4 is insulin-induced glucose uptake (Lizunov et al., 2013; Salt and Hardie, 2017). AMPK activation increases GLUT4 transfer to the plasma membrane and facilitates glucose uptake. Meanwhile, AMPK activation can increase PGC-1 expression to elevate GLUT4 expression (Fulco and Sartorelli, 2008). Given the effect of sodium butyrate on glucose metabolism and insulin resistance, we measured the levels of p-AMPK and GLUT4 in the liver and adipose tissue from sodium butyrate-treated C57BL/6J mice. As expected, we observed an increase in p-AMPK and GLUT4 levels in adipose tissue.

Butyrate is mostly produced by intestinal endothelium, and the concentration of butyrate is about 1–3 μM in the blood (Kasubuchi et al., 2015); thus, a metabolite derived from the interaction between butyrate and host could be responsible for improving MS. Previous studies have indicated that butyrate can stimulate intestinal epithelial cells to secrete glucagon-like peptide 1, thus increasing insulin sensitivity (Yadav et al., 2013). In our study, we observed that butyrate could increase the levels of α-linolenate, all-trans-retinal, resolvin E1, and leukotriene. The differential metabolic pathways are abundant in resolvin E1 biosynthesis, histidine degradation, lipoxin biosynthesis, and leukotriene biosynthesis. Resolvin E1 is a specialized pro-resolving lipid mediator, and it is linked to metabolic dysregulation and the immune system in type 2 diabetes. Resolvin E1 can resist LPS and TNFα induction of ERV1 overexpression and diabetic overexpression activating phagocytosis and resolution signals in human neutrophils (Freire et al., 2017). Thus, butyrate-induced resolvin E1 biosynthesis could be a potential mechanism through which butyrate can reduce unexplained inflammation. Lipoxin can attenuate obesity-induced adipose inflammation and alter the adipose M1/M2 ratio, promoting HFD-induced MS (Borgeson et al., 2015). However, we did not find any studies on the effect of butyrate on resolvin E1 and lipoxin metabolism for improving MS. We speculated that butyrate-induced biosynthesis of resolvin E1 and lipoxin could be a new pathway through which butyrate can reduce unexplained inflammation.

In conclusion, our study demonstrated that HFD increases the relative abundances of f_*Lachnospiraceae*, f_*Rikenellaceae*, and f_*Paraprevotellaceae* related to the levels of choline and BCAAs, which play a crucial role in the pathological process of atherosclerosis. Oral butyrate supplement could resist HFD-induced increase in f_*Lachnospiraceae*, f_*Rikenellaceae*, and f_*Paraprevotellaceae*. Meanwhile, oral butyrate supplement can regulate resolvin E1 biosynthesis, histidine degradation, lipoxin biosynthesis, and leukotriene biosynthesis, thus providing a new pathway through which butyrate can reduce unexplained inflammation related to CVD. Therefore, an oral supplement of the natural fermentation product butyrate can be a potential strategy for preventing CVD.

## Data Availability

The data that support the findings of this study are openly available in [gene bank] at https://www.ncbi.nlm.nih.gov/genbank, reference number [MN059945 - MN060968]. Accession numbers can be found in the **Supplementary Material**.

## Ethics Statement

The animal study was reviewed and approved by Ethics Committee of Institute of Materia Medica, Chinese Academy of Medical Sciences & Peking Union Medical College.

## Author Contributions

Conception and design of the study: FG and H-bZ; acquisition of data: FG, Y-WL, JL, and J-MC; analysis and interpretation of data: J-mH, X-ZR, and H-bZ.

## Funding

This work was supported by funding support from The Drug Innovation Major Project 2018ZX09711001-003-011, CAMS Innovation Fund for Medical Sciences 2016-I2M-1-009, and the National Natural Sciences Foundation of China (NSFC) (grant number 91539126).

## Conflict of Interest Statement

The authors declare that the research was conducted in the absence of any commercial or financial relationships that could be construed as a potential conflict of interest.

The reviewer YL declared a shared affiliation, with no collaboration, with several of the authors, FG, Y-WL, JL, J-MC, J-MH, and H-BZ, to the handling editor at the time of the review.
